# An Identity Management Scheme Based on Multi-Factor Authentication and Dynamic Trust Evaluation for Telemedicine

**DOI:** 10.3390/s25072118

**Published:** 2025-03-27

**Authors:** Yishan Wu, Mengxue Pang, Jianqiang Ma, Wei Ou, Qiuling Yue, Wenbao Han

**Affiliations:** 1 School of Cyberspace Security (School of Cryptology), Hainan University, Haikou 570228, China; 20223004988@hainanu.edu.cn (Y.W.); 24120854120002@hainanu.edu.cn (M.P.); ouwei@hainanu.edu.cn (W.O.); yueqiuling@hainanu.edu.cn (Q.Y.); 994338@hainanu.edu.cn (W.H.); 2 Laboratory for Advanced Computing and Intelligence Engineering, Wuxi 214100, China; 3 Jiangsu Variable Supercomputer Technology Co., Ltd., Wuxi 214100, China

**Keywords:** telemedicine, multi-factor authentication, ShangMi cryptographic algorithms, dynamic trust evaluation

## Abstract

Telemedicine diagnosis has become a more flexible and convenient way to receive diagnoses, which is of great significance in enhancing diagnosis, cutting costs, and serving remote users. However, telemedicine faces many security problems, such as the complexity of user authentication, the balance of the existing biometric factor authentication scheme, the unpredictability of user behavior, and the difficulty of unified authentication due to the differences in the security standards and authentication mechanisms of different trust domains, which affect the sustainable development of telemedicine. To address the above issues, this paper presents an identity management scheme based on multi-factor authentication and dynamic trust evaluation for telemedicine. Its authentication combines iris recognition for secure biometric verification, smart cards for encrypted credential storage, and static passwords for supplementary verification, addressing scenarios like facial coverage in medical settings. The scheme dynamically adjusts authentication based on attack rates, login anomalies, and service durations. By integrating ShangMi cryptographic algorithms and blockchain, it optimizes performance, achieving 35% lower communication overhead than previous protocols. A security analysis shows it resists impersonation, man-in-the-middle, and password modification attacks while preserving user anonymity. System evaluation meets authoritative standards, validating its practicality. This scheme balances security and efficiency, providing a strong basis for telemedicine’s long-term viability.

## 1. Introduction

Telemedicine is a medical service model that realizes remote diagnosis, treatment, and health monitoring between medical institutions and patients through the use of electronic communication technology and computer technology. At present, with the progress of the e-medical industry, telemedicine has gradually evolved from early telephone consultations to the stage of high-definition video consultations, remote surgery, and other high-end applications. Telemedicine enhances the quality and efficiency of medical services and also has significant potential to cut medical costs and fulfill the medical requirements of remote patients. However, the development of telemedicine does not rely on a single technical tool and requires a comprehensive, integrated, and efficient technical platform to support its complex application scenarios.

In telemedicine, patients can communicate with their doctors over the Internet to receive medical services [1]. Patients can obtain health data through wearable sensors and, with the help of mobile devices, send these data to the cloud database for doctors to access later. Meanwhile, doctors can conduct real-time consultations for patients in other places through remote cameras, smart audio, and other devices, and can even conduct remote surgery for patients through sophisticated smart operating tables.

However, while telemedicine brings great convenience to people’s lives, it also faces many security problems [2]. First, telemedicine accommodates diverse user groups (e.g., doctors, patients, and managers) with varying access privileges, necessitating granular authentication methods that balance security and usability. For instance, doctors require high-level access to medical records while patients need restricted view-only permissions. This diverse user authentication is more complicated [3]. Second, existing biometric schemes (e.g., fingerprint and face recognition) struggle to reconcile accuracy with convenience. In medical settings, facial coverage (e.g., masks) or device unfamiliarity among elderly patients often hinders verification, requiring supplementary factors like smart cards or passwords to ensure usability without compromising security. Meanwhile, user behavior patterns are inherently dynamic and influenced by external factors. Real-time trust assessments must analyze metrics like malicious attack rates and login anomalies, but this introduces computational complexity due to the need for continuous behavioral data collection and analysis. In addition, telemedicine involves the interoperation of multiple healthcare trust domains, such as the hospital domain and the patient domain, and the security standards and authentication mechanisms may differ between different trust domains, increasing the difficulty of unified authentication.

To address these issues, we propose an identity management scheme based on multi-factor authentication and dynamic trust evaluation for telemedicine. Specifically, we design and optimize the authentication scheme using a multi-technology fusion of authentication methods (including high-accuracy iris recognition [4], the secure storage of smart cards, and static password-assisted authentication [5]) to construct a three-factor system. Meanwhile, we established a dynamic trust evaluation system based on behavioral analysis by introducing a dynamic selection mechanism. This scheme not only improves data access control but also improves the security and convenience of authentication to facilitate the solution to the authentication needs of different types of users to prevent data leakage due to various factors, to enhance user trust and application scope, and to enhance the overall security of telemedicine.

The main contributions of this study are listed as follows:

- A three-factor authentication scheme using an iris, smart card, and static password is designed. The iris recognition has high uniqueness and accuracy, the smart card can securely store user information, and the static password also plays a supplementary role as a traditional authentication method. This combination can greatly improve the security of authentication and is more suitable for hospital personnel authentication when the face may be covered by protective equipment and when some patients are less familiar with the operation of smart devices.
- We introduce a dynamic selection mechanism for authentication factors and a dynamic trust evaluation mechanism based on user behavior analysis [6,7]. The system can automatically select appropriate combinations of authentication factors based on different user scenarios and equipment environments, making the authentication process more flexible and efficient and realizing a dynamic balance between authentication convenience and security. At the same time, the trust level is determined by a real-time evaluation of user behavior through the collection of a variety of data, which further enhances the security of telemedicine user authentication and ensures the secure and reliable operation of telemedicine.
- A scheme analysis reveals that the proposed scheme can effectively fend off common security threats including impersonation attacks, man-in-the-middle attacks, and static password modification attacks. Meanwhile, it ensures user anonymity and comprehensively protects user privacy and data security. Moreover, in the actual operation of telemedicine, it can meet the dual needs of the system for security and efficiency, providing solid support for the sustainable development of telemedicine services.

The rest of this paper is organized as follows. In Section 2, we reviewed some related works and analyzed the limitations of existing solutions. In Section 3, we explained the overall architecture and core modules of the system scheme. In Section 4, we first analyzed the correctness and security of the proposed scheme. Subsequently, we discussed the advantages of it in terms of authentication accuracy and communication. Finally, we discussed the function and computational costs of the scheme through a theorem-proving performance analysis. In Section 5, we conducted a system evaluation of the proposed solution. In Section 6, we concluded this paper and offered a perspective on upcoming research.

## 2. Related Work

In recent years, with the rapid development of telemedicine technology, new devices, technologies, and ways of sharing information ensure a more convenient and better life, which provides an efficient and convenient mode of communication between remote users and healthcare professionals. To guarantee the security of these communications, user authentication plays a vital role. Consequently, numerous researchers have put forward relevant user authentication schemes. In 2011, Das et al. [8] put forward an efficient biometric-based remote user authentication scheme. This scheme ingeniously utilized smart cards to achieve strong authentication and mutual authentication, laying the foundation for subsequent research. In 2012, An et al. [9] deeply analyzed the scheme proposed by Das et al. and discovered security vulnerabilities. They promptly proposed an enhanced scheme, effectively overcoming the security weaknesses of the original scheme and further enhancing the security of mutual authentication between users and servers. In the same year, Wu et al. [10] proposed an authentication scheme for mobile devices in a telecare medical information system, introducing a two-factor authentication using smart cards and passwords for telemedicine. They added the concept of pre-computation in the communication process to optimize computational efficiency. However, He et al. [11] found that Wu et al.’s scheme was vulnerable to risks such as insider attacks, impersonation attacks, and stolen smart card attacks, and proposed an improved version. Nevertheless, the improved scheme still had deficiencies in resisting brute-force password attacks. In 2014, after conducting in-depth analyses of the schemes proposed by Sood et al. [12] and Song et al. [13], Chen et al. [14] discovered problems in aspects such as mutual authentication and key security. Therefore, Chen et al. proposed an improved smart-card-based password authentication and key agreement scheme. This scheme achieved the functions of mutual authentication and protection against stolen smart card attacks, but was lacking in password updates and the prevention of insider attacks. In 2015, Das et al. [15] once again proposed a new smart-card-based robust and secure anonymous biometric remote user authentication scheme. This scheme used fuzzy extractors, encryption techniques, and anonymization processing. While ensuring the accuracy of identity and the security of authentication, it supported local password changes and protection against replay attacks. However, this scheme still had issues such as not supporting user anonymity and being vulnerable to various attacks. In 2018, Wu et al. [16] further developed this idea and proposed a two-factor authentication scheme suitable for resource-constrained medical sensor devices, providing additional security for these devices. This scheme used wireless healthcare sensor networks to prevent tracking and verified its security through the Proverif tool. However, it did not provide forward secrecy and had a high communication cost. In the same year, Challa et al. [17] proposed a provably secure three-factor user authentication and key agreement protocol for wireless medical sensor networks. This scheme improved security by using Elliptic Curve Cryptography (ECC) and supported the addition of dynamic sensor nodes, the update of passwords and biometrics, and the revocation of smart cards. It also conducted a security analysis through the ROR model and BAN logic, proving the effectiveness of the protocol in resisting various attacks and further enhancing the security protection level of the telemedicine system. However, this scheme was not secure against impersonation attacks and also had high computational and communication costs. In 2019, Barman et al. [18] proposed a three-factor authentication scheme for e-healthcare in a multi-server environment using fuzzy commitments. In 2020, Ali et al. [19] pointed out that it had various security risks. Ali et al. then proposed a new scheme. Although some problems were solved, there was still a risk of masquerade attacks and a lack of mutual authentication. In 2023, Gupta et al. [20] proposed a machine learning and smart-card-based two-factor authentication scheme for TMIS. This scheme integrates ML, nonce checks, and smart card blocking to prevent unauthorized access. While ensuring anonymity and AVISPA-validated attack resistance, it incurs ML overhead and smart card dependency, posing latency risks in real-time telecare. In 2024, Rao et al. [21] proposed L2FA-ADA-secure U-WSNs in eHealth with lightweight two-factor auth and anonymous data access, ensuring privacy and efficiency. Validated via security analysis and experiments, it resists attacks and outperforms peers. However, looking at these research results as a whole, most of them adopted single-factor or partial-factor authentication methods. For the complex medical environment and diverse user groups, it is difficult to strike a balance between authentication security and convenience.

More crucially, in the context of telemedicine, there is a severe scarcity of research integrating identity authentication with dynamic trust evaluation. The telemedicine environment is complex and ever-changing, and user behavior is uncertain. A single form of identity authentication cannot promptly address potential risks. However, organically combining identity authentication with dynamic trust evaluation can more accurately judge the authenticity and security of user identities and adjust authentication strategies and access rights in real time. Currently, though, relevant research is only in its infancy. Most studies merely focus on identity authentication technology itself or simply mention the concept of trust evaluation, lacking continuous dynamic monitoring and an in-depth analysis of user behavior.

In contrast, the scheme proposed in this paper has significant advantages. First, the scheme adopts a three-factor authentication method combining iris recognition, smart cards, and static passwords, fully utilizing the high precision of iris recognition, the secure storage of smart cards, and the auxiliary verification function of static passwords, which greatly improves the security of authentication and better adapts to situations where the face of hospital personnel may be covered by protective equipment during authentication and situations involving some patients who are not familiar with the operation of smart devices. Second, the introduction of the re-authentication mechanism and the dynamic trust evaluation mechanism based on user behavior analysis enables the system to acutely capture changes in user behavior, promptly adjust authentication strategies, and access rights. This effectively defends against various attacks and comprehensively safeguards user privacy and data security. Finally, it also shows advantages in terms of performance, which can further reduce time and communication overhead, improve system operation efficiency, and provide better authentication services for telemedicine.

## 3. System Solution

### 3.1. Overall Architecture

**Figure 1:**
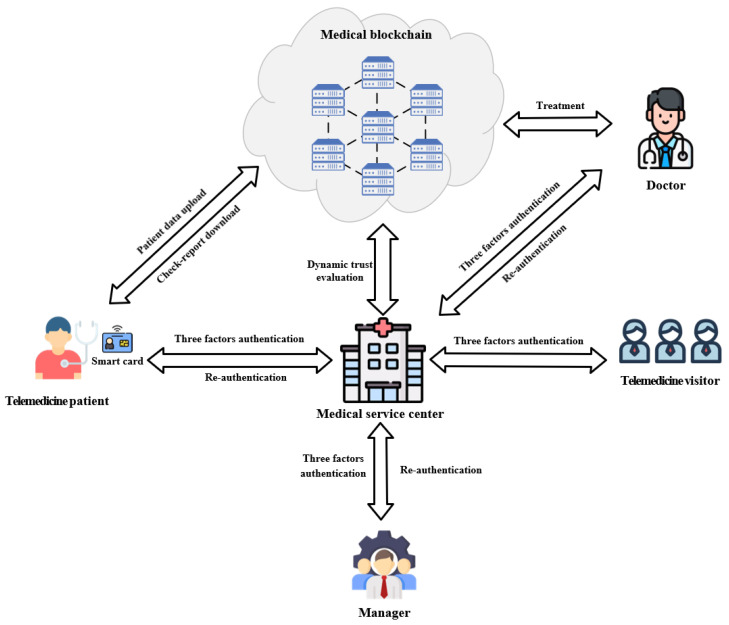
Overall architecture.

The overall architecture of this solution is shown in Figure 1. In this system, a Fabric consortium blockchain is adopted to implement the blockchain-based data-sharing platform. The uploading, querying, and updating of authentication information are achieved through the deployment of smart contracts. The medical service centers, medical sensors, etc., serve as participants to form a collaborative consortium of the blockchain network, leveraging the distributed ledger technology of blockchain to break the medical information silos. Entities interact with each other via secure communication protocols to jointly complete the identity authentication and management of telemedicine users, ensuring the security and integrity of information transmission. The proposed system model mainly includes the following types of entities:

**Medical service center:** Medical service centers offer remote medical services through various facilities, focusing on improving healthcare, reducing costs, and meeting users’ needs. It manages user registration and authentication and uses smart cards for three-factor authentication. During user interactions, in registration, it processes patient data under smart contract supervision. In authentication, it checks requests and smart contracts determine further steps. For visitors, smart contracts review and authorize. With medical blockchain, the service center sends encrypted data on registration and obtains a key. In daily operations, it uploads user data, and smart contracts support trust and permission management and provide data as needed.

**User entity:** Includes all objects that receive telemedicine services and have access to the medical data system. Four major groups are included in this program: doctors, managers, patients, and other telemedicine visitors.

- *Doctor*: As medical service providers, they interact with the medical service center in real time when accessing patient data and performing operations. The center obtains requests, smart contracts verify rights, and if passed, the center fetches data. Doctors’ behavior data are uploaded, and smart contracts analyze it, adjusting trust levels and access rights if there is abnormal behavior.
- *Manager*: Managers manage the system, including user and data management, and interact with the medical service center in real time. When allocating user permissions, their instructions go to the center. Smart contracts verify identities and authorities, and the center updates permission settings in real time and reports back results.
- *Telemedicine patient*: Patients interact with the medical service center in real time during registration, record viewing, and communication with doctors. At registration, their info goes to the center, and smart contracts verify it for proper storage. When viewing records, the center obtains requests, smart contracts check access rights, and the center fetches and returns data. For doctor communication, the center relays info while smart contracts safeguard its security and privacy.
- *Telemedicine visitor*: This includes third-party service orgs and patients’ family members. When applying for specific data, their authorization apps to the medical service center are received in real time. Smart contracts review apps per security and permission rules. After approval, during smart card and static password authentication, the info is sent to the center for verification. Smart contracts check its accuracy. If verified, visitors obtain access rights immediately.

**Medical blockchain [22]:** This consists of the medical information database used by the medical service center and the trusted server. The database is for user entities to upload and download data. The trusted server offers access control for dynamic three-factor authentication and dynamic trust evaluation when users download remote medical data. The medical service center uploads user registration, authentication, and behavioral data to the medical blockchain in real time. Blockchain smart contracts verify and store these data per preset rules for authenticity and integrity. When the medical service center needs to verify user permissions or obtain trust evaluation results, smart contracts quickly retrieve relevant data and give real-time feedback. When users download remote medical data, the trusted server conducts real-time dynamic three-factor authentication and trust evaluation. The smart contract determines the user’s trust level based on historical behavior and current operations. If the user meets the access requirements, the corresponding access rights are automatically granted. If the trust level is low or there are risks, the user is required to perform additional authentication steps or have their access rights restricted.

**Figure 2:**
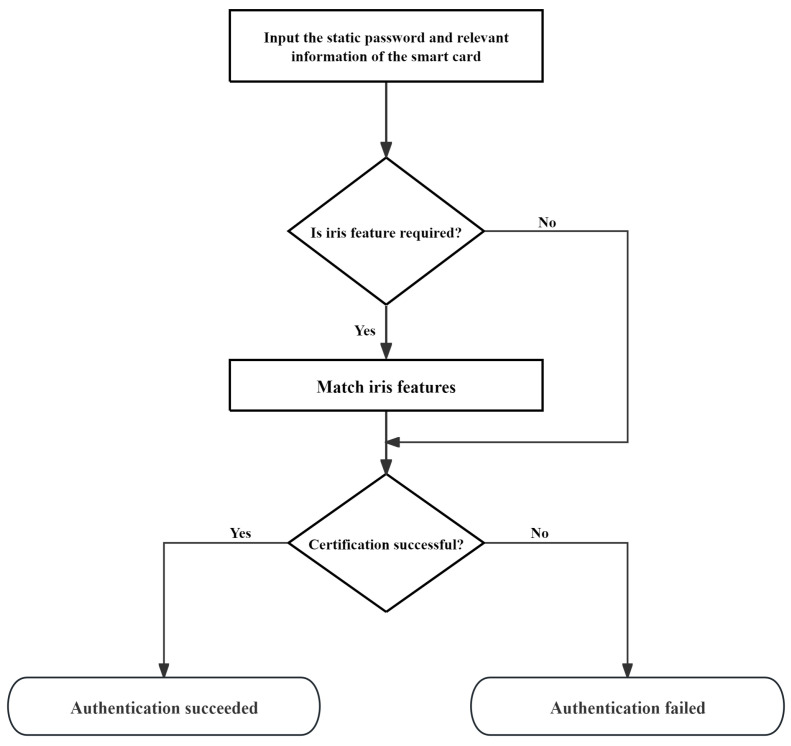
The flowchart of the patient authentication phase.

This paper proposes an identity management scheme based on multi-factor authentication and dynamic trust evaluation for telemedicine, whose workflow mainly includes two phases of registration and authentication. The flowchart of the patient authentication phase is shown in Figure 2. The specific description of re-authentication is located in Section 3.2.2.

The entire process, from the initial settings in the registration phase to the multiple verifications in the daily authentication, to the risk assessment and response based on user behaviors, and the secure handling of medical sensor data, ensures the security, privacy protection, and efficient operation of telemedicine.

### 3.2. Core Modules

The meaning of the symbols is shown in Table 1.

#### 3.2.1. Three-Factor Authentication

Our scheme adopts a three-factor authentication method combining iris recognition, smart cards, and static passwords and incorporates the state secret algorithm to enhance security, in which SM2 is used for asymmetric encryption and SM3 is used for hashing, and adopts a dynamic and flexible authentication factor scheme to adapt to different situations to balance the authentication convenience and security.

**Registration stage:** The registration stage consists of three components, including medical service center registration, the registration of user entities, and medical sensor registration.

- *Medical service center registration*: The medical service center MSC generates the registration random number $R_{MSC}$ and calls SM3 for hashing to generate the current timestamp $T_{1}$ and uses the SM2 algorithm to generate the public key $PU_{MSC}$ of the medical blockchain to send the relevant information $\left\{MSCid,R_{MSC1},T_{1}\right\}$ encrypted as C1 by SM2 to the medical blockchain. The medical blockchain receives the request and uses SM2 to decrypt $PR_{MCS}$, after which it generates the current timestamp $T_{1}^{\prime }$ and verifies whether $|T_{1}^{\prime }-T_{1}|\leq \Delta T$ is established; if it passes, it checks whether $|T_{1}^{\prime }-T_{1}|\leq \Delta T$ is registered, and if it is not registered, it selects $K_{MSC-MC}\in L$ and encrypts C2 using the SM2 encryption algorithm and the key $K_{MSC}=h(MSCid∥R_{MSC1}∥T_{1})$ to obtain C2 and sends C2 to the medical service center. The medical service center calculates $K_{MSC}$, decrypts C2, generates the current timestamp $T_{2}^{\prime }$, verifies whether $|T_{2}^{\prime }-T_{2}|\leq \Delta T$ is established, and if established, saves it as the data encryption key.
- *Registration of user entities*: User $U_{i}$ uses the user terminal in the secure environment of the medical service center to enter account number $Uid_{i}$, login key $Upw_{i}$, and iris characteristics $BIO_{i}$. The user terminal generates the registration random number $R_{UR1}$, uses the gen() function to generate the data required for iris detection to compute $\left(\sigma_{i},\tau_{i}\right)=Gen\left(BIO_{i}\right)$, and then uses the SM3 hash algorithm to compute $UR_{UR1}=R_{UR1}⊕h\left(Uid_{i}∥Upw_{i}\right)$, $M_{i}=h(Uid_{i}∥Upw_{i}∥\sigma_{i}∥R_{UR1}∥MSCid)$, and $N_{i}=h(Uid_{i}∥Upw_{i}∥\tau_{i}∥R_{UR1}∥MSCid)$ to generate the current timestamp $T_{3}$ and provides $\left\{Uid_{i},Mi,Ni,UR_{UR1},T_{3}\right\}$ to the medical service center. The medical service center receives the enrollment request, generates the current timestamp $T_{3}^{\prime }$, verifies whether $|T_{3}^{\prime }-T_{3}|\leq \Delta T$ is valid, generates the enrollment random number $R_{UR2}$, selects the smart card numbered Cid, and calls the SM3 hash algorithm to calculate $A_{i}=h(Uid_{i}∥M_{i}∥MSCid∥Cid∥R_{UR2})$, $B_{i}=h(Uid_{i}∥N_{i}∥MSCid∥Cid∥R_{UR2})$, $UA_{i}=A_{i}⊕M_{i}$, $UB_{i}=B_{i}⊕N_{i}$, $I_{U}=h\left(UR_{UR1}∥MSCid\right)$, $HA_{i}=A_{i}⊕I_{U}$, and $HB_{i}=B_{i}⊕I_{U}$. The $\left\{I_{U},HA_{i},HB_{i},T_{3},Li\right\}$ is stored in the database and the $\left\{U_{A_{i}},U_{B_{i}}\right\}$ is encrypted by the SM2 algorithm stored in the smart card and issued to the user. After the user receives the information, $\left\{UR_{UR1}\right\}$ is stored in the smart card, and the smart card saves $\tau_{i},UA_{i},UB_{i},UR_{UR1}$.
- *Medical sensor registration*: The medical sensor generates the registration random number $R_{SR1}$, calls the SM3 hash algorithm to calculate $SSid_{j}=h(R_{SR1}∥Sid_{j}∥MSCid)$ and $SR_{SR1}=R_{SR1}⊕h\left(Sid_{j}∥MSCid\right)$, obtains and generates the current timestamp $T_{4}$, and sends $\left\{Sid_{j},SR_{SR1},SSid_{j},T_{4}\right\}$ to the gateway through a secure channel. The gateway sends the information to the medical service center. The medical service center receives the message, generates the current timestamp $T_{4}^{\prime }$, verifies whether $|T_{4}^{\prime }-T_{4}|\leq \Delta T$ is valid, verifies the freshness of the message, and if the judgment is successful, generates the registered random number $R_{SR2}$ and uses the SM3 algorithm to calculate $C_{j}=h(SR_{SR1}∥SSid_{j}∥R_{SR2}∥MSCid)$, $HC_{j}=C_{j}⊕h\left(MSCid∥SSid_{j}\right)$, and $SC_{j}=C_{j}⊕h\left(MSCid∥SR_{SR1}\right)$; generates the current timestamp $T_{5}$; stores $\left\{Sid_{j},HC_{j},SSid_{j}\right\}$ in the database; and sends $\left\{T_{5},HC_{j}\right\}$ to the medical sensor. The medical sensor receives the message, generates the current timestamp $T_{5}^{\prime }$, verifies whether $|T_{5}^{\prime }-T_{5}|\leq \Delta T$ is valid, and if the judgment is successful, then invokes the SM3 hash algorithm to calculate $SC_{j}=C_{j}⊕h\left(MSCid∥R_{SR1}\right)$, and $\left\{SR_{SR1},SC_{j},SSid_{j}\right\}$ will be stored locally.

**Authentication phase:** In this phase, the user inserts the smart card into the terminal device, the terminal device acquires $\left\{Cid,UA_{i},UB_{i},UR_{UR1},\tau_{i}\right\}$, and the user name $Uid_{i}$ and key $Upw_{i}$ entered by the user generates the current timestamp $T_{6}$ using the SM3 algorithm and sends $\left\{T_{6},UR_{UR1}\right\}$ to the medical service center. The medical service center receives the information, generates the current timestamp $T_{6}^{\prime }$, verifies whether $|T_{6}’-T_{6}|\leq \Delta T$ is valid, if the judgment is successful, calls the SM3 hash algorithm to calculate $I_{U}=h\left(UR_{UR1}∥MSCid\right)$, searches for table entries in the database with $I_{U}$ as the index to obtain the corresponding $\left\{T_{3},L_{i}\right\}$, checks whether $T_{6}-T_{3}$ is outdated, and verifies whether the value of $L_{i}$ is “3” if the verification is passed; if it is “3”, reject the user request, and if the verification passes, then temporarily store $\left\{UR_{UL},UA_{i},UB_{i},HR_{UR2}\right\}$ and generate the current timestamp $T_{7}$ and send $\left\{T_{7},L_{i}\right\}$ to the user. The user terminal receives the information and generates the current timestamp $T_{7}^{\prime }$, verifies whether $|T_{7}^{\prime }-T_{7}|\leq \Delta T$ is valid, and if the judgment is successful, calls the SM3 hash algorithm to calculate $R_{UR1}=UR_{UR1}⊕h\left(Uid_{i}∥Upw_{i}\right)$; the terminal generates a random number $R_{UL1}$ and calls the SM3 algorithms to generate the current timestamp $T_{8}$. The user inputs the target device number $Sid_{j}$, calls the SM3 hash algorithm to calculate $USid_{j}=Sid_{j}⊕h\left(Uid_{i}∥R_{UL1}\right)$ and $UR_{UL1}=R_{UL1}⊕h\left(Cid∥MSCid\right)$, and requests the user to provide the authentication factor according to $L_{i}$, and when it is “1”, calculate $M_{i}=h(Uid_{i}∥Upw_{i}∥R_{UR1})$ and $A_{i}=MA_{i}⊕M_{i}$, $V0_{i}^{\prime }=h\left(A_{i}∥R_{UL1}\right)$; when it is “2”, the terminal enters the user’s iris feature specimen $BIO_{i}^{\prime }$ and calls the hash algorithm to calculate $\sigma_{i}=Rep\left(BIO_{i}^{\prime },\tau_{i}\right)$, $N_{i}=h(Uid_{i}∥Upw_{i}∥\sigma_{i}∥R_{UR1})$, $B_{i}=MB_{i}⊕N_{i}$, and $V0_{i}=h\left(B_{i}∥R_{UL1}\right)$. $\left\{T_{8},UR_{UL1},UR_{UR1},V0_{i}’,USid_{j}\right\}$ is sent to the medical service center for the temporary storage of $R_{UL1}$. The medical service center receives the information and generates the current timestamp to verify whether $|T_{8}’-T_{8}|\leq \Delta T$ is valid, and if successful, it calls the SM3 hash algorithm to calculate $A_{i}=HA_{i}⊕h\left(MSCid∥UR_{UR1}\right)$, $B_{i}=HB_{i}⊕h\left(MSCid∥UR_{UR1}\right)$, and $R_{UL1}=UR_{UL1}⊕h\left(Cid∥MSCid\right)$ and calculates $V0_{i}=h\left(A_{i}∥R_{UL1}\right)$ or $V0_{i}=h\left(B_{i}∥R_{UL1}\right)$ based on $L_{i}$ and verifies whether $V0_{i}’$ is equal to $V0_{i}$. If the verification is successful, it completes the verification of the user. The medical service center calculates $Sid_{j}=USid_{j}⊕h\left(Uid_{i}∥R_{UL1}\right)$ based on the $USid_{j}$ provided by the user and, using the SM3 hash algorithm, looks up and obtains $\left\{HC_{j},SSid_{j}\right\}$ in the medical service equipment table based on $Sid_{j}$, calculates $C_{j}=HC_{j}⊕h\left(MSCid∥SSid_{j}\right)$ and $V1_{j}=h(MSCid∥SSid_{j}∥C_{j})$, generates a random number $R_{UL2}$, generates a current timestamp $T_{9}$, and sends $\left\{T_{9},V1_{j}\right\}$ to the medical sensor for temporary storage of $\left\{T_{9},V1_{j}\;R_{UL1},R_{UL2}\right\}$. The medical sensor receives the information and first generates the current timestamp $T_{9}^{\prime }$, verifies that $|T_{9}’-T_{9}|\leq \Delta T$ is valid, and, if the judgment is successful, invokes the SM3 hash algorithm to calculate $R_{SR1}=SR_{SR1}⊕h\left(Sid_{j}∥MSCid\right)$, $C_{j}=SC_{j}⊕h\left(MSCid∥R_{SR1}\right)$, and $V1_{j}^{\prime }=h(MSCid∥SSid_{j}∥C_{j})$ and verify whether $V1_{j}^{\prime }$ and $V1_{j}$ are equal; if they are equal, the verification passes, the medical sensor generates $R_{UL3}$, calculates $V2_{j}=h(C_{j}∥HMSid∥SSid_{j})$ and $SR_{UL3}=R_{UL3}⊕h\left(C_{j}∥V1_{j}\right)$ by the SM3 hash algorithm, generates the current timestamp $T_{10}$, and sends $\left\{T_{10},V2_{j},SR_{UL3}\right\}$ to the medical service center. The medical service center receives the message and generates the current timestamp $T_{10}^{\prime }$, verifies whether $|T_{10}’-T_{10}|\leq \Delta T$ is valid, and if the judgment is successful, it calls the SM3 hash algorithm to calculate $C_{j}=HC_{j}⊕h\left(MSCid∥SSid_{j}\right)$ and $V2_{j}^{\prime }=h(C_{j}∥MSCid∥SSid_{j})$ and verifies whether $V2_{j}^{\prime }$ and $V2_{j}$ are equal. If the verification is successful, it passes the verification of the medical sensor and generates the current timestamp $T_{11}$ by using the SM3 hash algorithm to calculate $R_{UL3}=SR_{UL3}⊕h\left(C_{j}∥V1_{j}\right)$ and $K=h(T_{11}∥h\left(R_{UL1}\cdot R_{UL2}\cdot R_{UL3}\right))$ and calculates $V3_{i}^{\prime }=h\left(MR_{UR1}∥V0_{i}\right)$, $UK=R_{UL2}\cdot R_{UL3}$, $V4_{j}^{\prime }=h\left(SR_{UL3}|V2_{j}\right)$, and $SK=R_{UL1}\cdot R_{UL2}$, sends $\left\{T_{11},V3_{i}^{\prime },UK\right\}$ to the user, and sends $\left\{T_{11},V4_{j}^{\prime },K\right\}$ to the medical sensor. The user receives the message, generates the current timestamp $T_{11}^{\prime }$, and verifies that $|T_{11}’-T_{11}|\leq \Delta T$ is valid. If the judgment is successful, it calculates $V3_{i}=h\left(MR_{UL1}∥V0_{i}\right)$ using the SM3 hash algorithm, verifies that $V3_{i}$ is equal to $V3_{i}^{\prime }$, and if equal, the verification passes and calculates $K=h(T_{11}h\left(R_{UL1}\cdot UK\right))$. The medical sensor receives the message and also generates the current timestamp $T_{11}^{\prime }$ and verifies it using the SM3 hash algorithm. If the judgment is successful, it calculates $V4_{j}=h\left(SR_{UL3}V2_{j}\right)$, verifies that $V4_{j}$ is equal to $V4_{j}^{\prime }$, and if equal, the verification passes and calculates $K=h(T_{11}h\left(R_{UL3}\cdot UK\right))$.

#### 3.2.2. Dynamic Trust Evaluation

On the basis of three-factor authentication to determine the user’s identity, in order to enhance the security of telemedicine user authentication, we introduced dynamic trust evaluation based on user behavior analysis, which relies on some of the data collected during three-factor authentication, and is able to analyze the user’s behavior in depth further, assess the user’s risk level in real time, and ensure that telemedicine can be operated securely and reliably.

After considering multiple factors, we chose six key indicators for telemedicine security: malicious attack rate, attempted override rate, login system anomaly rate, access service duration, user operation habit stability, and sensitive data utilization rate. The malicious attack rate weighs threats by harm degree to warn users. The attempted override rate spots internal risks from unauthorized actions. The login system anomaly rate blocks illegal logins. The user operation habit stability flags abnormal behavior. The sensitive data utilization rate safeguards data confidentiality. Access service duration ensures proper user operation. These six indicators build a comprehensive trust model, ensuring reliable user trust evaluation and overall telemedicine security. The description of these indicators is listed as follows:

- *Malicious attack rate* [23]: The malicious attack rate refers to the frequency of malicious attacks (such as user impersonation attacks, man-in-the-middle attacks, static password guessing attacks, and other types of attacks that intentionally damage system security, steal information, or interfere with normal services) launched against the system within a specific period. It reflects the frequency of malicious attacks on the system. Let i denote the number of times the first malicious attack behavior is detected, and $n_{i}$ denotes the total number of accesses in the same period. The attempted transgression rate is calculated as follows:
  (1) $$T_{1}=\frac{\sum_{i=1}^{k}\omega_{i}\times n_{i}}{N_{t1}}$$
  where *k* is the number of types of malicious attacks, considering the different degrees of harm of different types of malicious attacks, where each type of malicious attack sets the corresponding weight factor $\omega_{i}$.
- *Attempted override rate*: Attempts to overstep the authority rate refer to the frequency of users attempting to access resources beyond their authorized scope or perform unauthorized operations within a certain period, including patients attempting to access the medical records of other patients, non-medical personnel attempting to perform medical operation-related commands, and ordinary users attempting to obtain system management privileges and other behaviors. It reflects the degree of users’ compliance with system privilege rules and the degree of potential security risk. Setting the number of times a user attempts to access unauthorized resources in a certain period for the *j*th operation category as $n_{j}$, the corresponding weight as $\omega_{i}$, and the total number of access attempts in the same period as $N_{t2}$, the malicious attack rate is calculated as follows:
  (2) $$T_{2}=\frac{\sum_{j=1}^{m}\omega_{i}\times n_{j}}{N_{t2}}$$
  where *m* is the number of operation categories involved in the user’s attempts to transgress authorization.
- *Login system anomaly rate* [24]: The login system anomaly rate is the frequency of authentication failures or errors due to various reasons within a certain period. This includes but is not limited to multiple login attempts with incorrect usernames or static passwords, frequent logins within a short period, logins from unusual geographic locations or IP addresses, login attempts with known malware or tools, and so on. Setting the number of anomalies detected by the login system in a given period as $N_{l}$ and the total number of login attempts in the same period as $N_{t3}$, the login system anomaly rate is calculated as follows:
  (3) $$T_{3}=\frac{N_{l}}{N_{t3}}$$
- *Access service duration* [25]: The access service duration is the average length of time from when a user successfully logs into the system to when they log out of the system or when the session times out. This period can reflect the user’s activity level and usage habits in the system, as well as the response time and performance issues of the service. Let the duration of the *i*th service access be $t_{i}$, the length of time from the beginning to the end of the request, and the total number of service accesses in a certain period is *N*; then, the access service duration is calculated as follows:
  (4) $$T_{4}=\frac{1}{N}\sum_{i=1}^{N}t_{i}$$
- *Stability of user operation habits* [26]: The stability of user operation habits refers to the degree of deviation between a user’s operation behaviors within a system and their past habits within a certain period of time. Suppose that within the investigation period, the number of deviations in the user’s operation behaviors from their past habits is $N_{k}$ (where *k* represents the type of operation, such as deviation in login time, deviation in login device, deviation in operation frequency, etc.), the weight of each operation type is $\omega_{i}$, and the total number of access attempts within the same time period is $N_{t3}$. Then, the calculation formula for the stability of user operation habits is
  (5) $$T_{5}=\frac{\sum_{k=1}^{m}\omega_{i}\times n_{k}}{N_{t5}}$$
  where *m* is the number of operation categories.
- *Sensitive data utilization rate*: The sensitive data utilization rate is used to measure the frequency at which users access and use sensitive data in the system, encompassing operations such as users accessing, downloading, and sharing sensitive data. Assume that the number of operations a user conducts on sensitive data within a certain period of time is $N_{r}$, and the total number of data operations within the same time period is $N_{t6}$. Then, the calculation of the utilization rate of sensitive data is as follows:
  (6) $$T_{6}=\frac{N_{r}}{N_{t6}}$$

For users logging into the system for the first time, due to the lack of sufficient behavioral data for a comprehensive assessment, we give a relatively high initial user trust value *Q* (*Q* = 1) to enhance the ease of authentication and ensure that users can use basic healthcare services without any problems. As the user’s activities in the system increase and the behavioral data accumulate, we will pay close attention to those behavioral signs that may lead to an increased risk level, and the trust value will drop rapidly in the case of frequent abnormal behaviors (e.g., multiple abnormal samples in a short period). The formula for calculating the comprehensive trust value is as follows:

(7) $$T=\varphi_{1}\left(1-T_{1}\right)+\varphi_{2}\left(1-T_{2}\right)+\varphi_{3}\left(1-T_{3}\right)+\varphi_{4}\left(1-T_{4}\right)+\varphi_{5}\left(1-T_{5}\right)+\varphi_{6}\left(1-T_{6}\right)$$

where $T_{1}$, $T_{2}$, $T_{3}$, $T_{4}$, $T_{5}$, and $T_{6}$ denote the malicious attack rate, attempted transgression rate, login system anomaly rate, access service duration, stability of user operation habits, and sensitive data utilization rate, respectively, and the weights of each indicator are set to be $\varphi_{1}$, $\varphi_{2}$, $\varphi_{3}$, $\varphi_{4}$, $\varphi_{5}$, and $\varphi_{6}$, respectively, and $\varphi_{1}+\varphi_{2}+\varphi_{3}+\varphi_{4}+\varphi_{5}+\varphi_{6}=1$.

In real-world scenarios, the six key indicators for telemedicine security we chose are based on extensive research and analysis on actual medical data. For example, the malicious attack rate is calculated based on the historical data of known malicious attacks in similar telemedicine systems. This data-driven approach ensures that our dynamic trust evaluation mechanism can accurately assess the risk level of users. When a malicious attack is detected, the system not only blocks the attacker’s access but also alerts the system administrators. They can then take further actions, such as investigating the source of the attack and strengthening the security of the system. In terms of handling other security threats like attempted override of authority, the system logs every access attempt and cross-checks it with the user’s authorized privileges. If an unauthorized access attempt is detected, the system immediately locks the user’s account and initiates an investigation. These measures have been proven effective in simulations and pilot studies, and we are confident they will be effective in real-world applications.

By analyzing and modeling user and entity behaviors, a triple baseline is constructed. Based on the behavioral baseline of different risks, the trust level is divided into four categories, namely untrustworthy, basic trustworthy, general trustworthy, and high trustworthy. Based on the behavioral scores of users and entities falling into different trust value intervals, users can be defined into four classes, which are low-risk users, normal users, medium-risk users, and high-risk users. According to the results of the dynamic trust evaluation, the telemedicine visitor is graded in terms of authority, and the trust level is divided, as shown in Table 2.

**Authentication policy and factor updates:** Depending on the risk level of the user, we will take appropriate measures to securiguard the security of the system. For normal and low-risk users, as they are assessed as having a low risk, the subsequent authentication process can be simplified, and they will continue to operate as normal, with no additional steps required to complete authentication by simply providing a smart card. For medium-risk users, the medical service center will immediately initiate the re-authentication process and lock the uploading of data access requests for this level of users as well as the data of the medical sensors employed by the users at the same time. For high-risk users, more stringent measures will be taken to directly block them from further accessing the system until a higher level of authentication is completed and the authentication factor is updated.

The process of updating the identification factor is as follows. The banned user $U_{i}$ inputs the identification factor update request to the user terminal, inserts the smart card into the terminal device, acquires $\left\{Cid,MA_{i},MB_{i},UR_{UR1}\right\}$, inputs the user name $Uid_{i}$ and key $Upw_{i}$, generates the current timestamp $T_{12}$ with the key update random number $R_{UU1}$, and the terminal enters the user’s iris feature specimen $BIO_{i}^{\prime }$, $U_{UR1}=UR_{UR1}⊕h\left(Uid_{i}∥Upw_{i}\right)$; calculates $\sigma_{i}=Rep\left(BIO_{i}^{\prime },\tau_{i}\right)$, $N_{i}=h(Uid_{i}∥Upw_{i}∥\sigma_{i}∥R_{UR1})$, $B_{i}=MB_{i}⊕N_{i}$, and $V5_{i}^{\prime }=h\left(B_{i}∥R_{UU1}\right)$; sends $\left\{T_{12},UR_{UU1},V5_{i}^{\prime }\right\}$ to the medical service center; and stores it temporarily as $R_{UU1}$. The medical service center generates the current timestamp $T_{12}^{\prime }$ and verifies whether $|T_{12}’-T_{12}|\leq \Delta T$ is valid; if the judgment is successful, then it calculates $B_{i}=HB_{i}⊕h\left(MSCid∥UR_{UR1}\right)$, $R_{UU1}=UR_{UR1}⊕h\left(Cid∥MSCid\right)$, and $V5_{i}=h\left(B_{i}∥R_{UU1}\right)$, and verifies whether $V5_{i}^{\prime }$ is equal to $V5_{i}$. If the verification is successful, then it completes the verification of the user, generates the key update random number $R_{UU2}$, generates the current timestamp $T_{13}$, calculates $V6_{i}^{\prime }=h(R_{UU1}∥MSCid∥Cid)$ and $HR_{UU2}=R_{UU2}⊕h(T_{13}∥R_{UU1}∥MSCid)$, and sends $\left\{T_{13},V6_{i}^{\prime },HR_{UU2}\right\}$ to the user terminal and temporary storage $R_{UU2}$. The user terminal receives the information, verifies that the current timestamp $T_{13}^{\prime }$ is generated, verifies whether $|T_{13}^{\prime }-T_{13}|\leq \Delta T$ is valid, calculates $V6_{i}=h(R_{UU1}∥MSCid∥Cid)$ if the judgment is successful, verifies whether $V6_{i}^{\prime }$ is equal to $V6_{i}$, and verifies that it is successful. If so, it calculates $R_{UU2}=HR_{UU2}⊕h(T_{13}∥R_{UU1}∥MSCid)$, and the user enters a new factor, the login key $Upw_{i}^{new}$, and enters the iris characteristics $BIO_{i}^{new}$. The user terminal generates the key update random number $R_{UR1}^{new}$ and calculates $\left(\sigma_{i}^{new},\tau_{i}^{new}\right)=Gen\left(BIO_{i}^{new}\right)$, $UR_{UU2}=R_{UU2}⊕h(T_{13}∥R_{UU1}∥MSCid)$, $UR_{UU1}^{new}=R_{UR1}^{new}⊕h\left(Uid_{i}∥Upw_{i}^{new}\right)$, $M_{i}^{new}=h(Uid_{i}∥Upw_{i}^{new}∥R_{UR1}^{new}∥MSCid)$, and $N_{i}^{new}=h(Uid_{i}∥Upw_{i}^{new}∥R_{UR1}^{new}∥MSCid)$; generates the current timestamp $T_{13}$; and provides $\left\{M_{i}^{new},N_{i}^{new},UR_{UR1}^{new},T_{13},UR_{UU2}\right\}$ to the medical service center. The medical service center receives the registration request; generates the current timestamp $T_{13}^{\prime }$; verifies whether $|T_{13}^{\prime }-T_{13}|\leq \Delta T$ is valid; generates the key update random number $R_{UR2}^{new}$; calculates $A_{i}^{new}=h(Uid_{i}∥M_{i}^{new}∥MSCid∥Cid∥R_{UR2}^{new})$, $B_{i}^{new}=h(Uidi∥N_{i}^{new}∥MSCid∥Cid∥R_{UR2}^{new})$, $UA_{i}^{new}=A_{i}^{new}⊕M_{i}^{new}$, $UB_{i}^{new}=B_{i}^{new}⊕N_{i}^{new}$, $HA_{i}^{new}=A_{i}^{new}⊕h\left(MSCid∥UR_{UR1}^{new}\right)$, $HB_{i}^{new}=B_{i}^{new}⊕h\left(MSCid∥UR_{UR1}^{new}\right)$, and $I_{U}^{new}=h\left(UR_{UR1}^{new}∥MSCid\right)$; stores $\left\{IU^{new},HA_{i}^{new},HB_{i}^{new},T_{13},L_{i}\right\}$ in the database; and $L_{i}$ is the user identification factor policy that is periodically updated from the medical blockchain. The calculation $R_{UU2}^{\prime }=UR_{UU^{\prime }2}⊕h(T_{13}∥R_{UU1}∥MSCid)$ verifies that $R_{UU2}^{\prime }$ and $R_{UU2}$ are equal, and $\left\{UA_{i}^{new},UB_{i}^{new}\right\}$ is sent to the user if the verification is successful. The smart card provided by the medical service center has a limitation of time; when the smart card expires, the user needs to re-visit the medical service center for re-registration. The user receives the information and re-deposits $\left\{UA_{i}^{new},UB_{i}^{new},\tau_{i}^{new},UR_{UR1}^{new}\right\}$ to the smart card.

#### 3.2.3. Re-Authentication

When the dynamic trust evaluation finds that the user level reaches medium risk, the medical service center will immediately start the re-authentication process with the following steps:

Step 1: The medical service center receives the updated $L_{i}$ from the medical blockchain and then re-authenticates the online user with increased risk and immediately locks the upload of the data access request of the risky user as well as the upload of the data of the medical sensors adopted by the user, encrypts the re-authentication message $MSG_{1}$ by the key *K* generated in the key negotiation stage, and then generates the current timestamp $T_{14}$ by using the SM3 algorithm, and sends the ${\left\{MSG_{1},T_{14},MSCid\right\}}_{k}$ to the user terminal.

Step 2: The user terminal receives the message and uses the SM3 algorithm to generate the current timestamp $T_{14}^{\prime }$, verifies whether $|T_{14}^{\prime }-T_{14}|\leq \Delta T$ is valid, decrypts the message, and requires the user to perform strong identity authentication. The user inserts the smart card into the terminal device, acquires $\left\{Cid,UA_{i},UB_{i},UR_{UR1},\tau_{i}\right\}$, enters the user name $Uid_{i}$ and the key $Upw_{i}$, enters the iris feature specimen $BIO_{i}^{\prime }$, and the terminal calculates $R_{UR1}=UR_{UR1}⊕h\left(Uid_{i}∥Upw_{i}\right)$ to generate the random number $R_{URL1}$. The terminal generates the current timestamp $T_{15}$ by calculating $USid_{j}=Sid_{j}⊕h\left(Uid_{i}∥R_{URL1}\right)$, $UR_{URL1}=R_{URL1}⊕h\left(Cid∥MSCid\right)$, $\sigma_{i}=Rep\left(BIO_{i}^{\prime },\tau_{i}\right)$, $N_{i}=h(Uid_{i}∥Upw_{i}∥\sigma_{i}∥R_{UR1})$, $B_{i}=MB_{i}⊕N_{i}$, and $V7_{i}^{\prime }=h\left(B_{i}∥R_{URL1}\right)$ through the SM3 algorithm and sends $\left\{T_{15},UR_{URL1},UR_{UR1},V7_{i}^{\prime }\right\}$ to the medical service center, which temporarily stores $\left\{R_{URL1}\right\}$.

Step 3: After the medical service center terminal receives the information, it uses the SM3 algorithm to verify the generation of the current timestamp $T_{15}^{\prime }$, verifies whether $|T_{15}^{\prime }-T_{15}|\leq \Delta T$ is valid, and verifies whether $|T_{15}^{\prime }-T_{14}|$ exceeds the time limit for re-authentication. If the verification is successful, it calculates $B_{i}=HB_{i}⊕h\left(MSCid∥UR_{UR1}\right)$, $R_{UL1}=UR_{UL1}⊕h\left(Cid∥MSCid\right)$, and $V7_{i}=h\left(B_{i}∥R_{URL1}\right)$ by the SM3 algorithms and verifies whether $V7_{i}^{\prime }$ is equal to $V7_{i}$. If the verification is successful, it completes the re-authentication of the user.

Step 4: The medical service center lifts the data access lock and the lock of relevant medical sensor data upload for the user who has passed the re-authentication and readjusts the risk level of the user.

**Figure 3:**
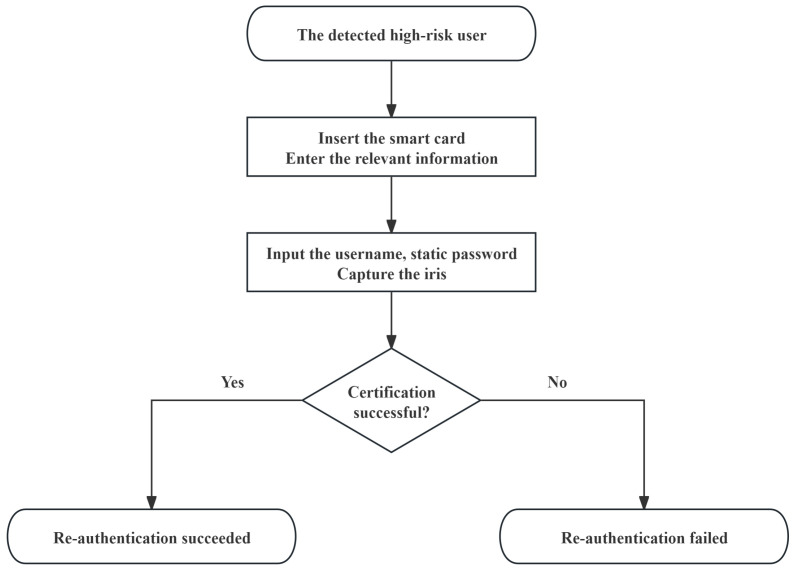
The flowchart of the patient re-authentication.

The flowchart of the patient re-authentication is shown in Figure 3.

Re-authentication is a complementary measure for user risk changes based on three-factor authentication and dynamic trust evaluation. When the dynamic trust evaluation detects an increase in the user’s risk, the medical service center determines the users who need to be re-authenticated based on the updated data provided by the medical blockchain and sends a notification to their terminals. The user is required to authenticate again through the three-factor authentication process. If the user passes the re-authentication, the restrictions on data access and sensor data upload will be lifted, and the user’s risk level will be readjusted; if not, stricter access restrictions and other security measures will be taken. This mechanism can effectively ensure that in the event of a change in the user’s risk, especially if the risk increases, the system can re-verify the user in a timely manner to protect the security and stability of the system.

## 4. Scheme Analysis

### 4.1. Correctness Analysis

The correctness of this subsection lies mainly in two aspects: the analysis of correctness in the registration phase and that in the user authentication phase.

#### 4.1.1. Correctness Analysis of the Registration Phase

**Medical service center registration:** The medical service center MSC generates the registration random number $R_{MSC1}$ and timestamp $T_{1}$ and uses $PU_{MCS}$ to encrypt the relevant information to send to the medical blockchain. The medical blockchain decrypts and performs time verification and registration status checks and then selects the key $K_{MCS}=h\left(MSCidR_{MCS1}T_{1}\right)$ and sends it to the medical service center. The medical service center decrypts and saves the data encryption key. The whole process ensures the secure transmission and correct processing of information and ensures that the registration information of the medical service center is accurately and correctly registered in the medical blockchain.

**User entity registration stage:** User $U_{i}$ enters the account number, $Uid_{i}$ login key $Upw_{i}$, and enters iris features $BIO_{i}$ in the secure environment of the medical service center. The user terminal generates relevant data and provides them to the medical service center. The medical service center receives the registration request and carries out operations such as time verification, random number generation, and smart card selection, and stores the relevant information in the database and smart card. The user receives the information and deposits part of the data into the smart card, which ensures complete and accurate storage of user registration information and provides a reliable basis for subsequent authentication.

**Medical sensor registration:** The medical sensor generates the registration random number $R_{SR1}$, calculates the relevant identification, and sends the information to the gateway through a secure channel, after which the gateway sends the information to the medical service center. The medical service center carries out time verification, message freshness verification, and other operations; stores the relevant information in the database; and sends it to the medical sensor. The medical sensor receives the message and then verifies and stores the data, which ensures that the registration information of the medical sensor is correctly configured in the medical service center and the sensor locally.

#### 4.1.2. Correctness Analysis of the User Authentication Phase

**Authentication and key negotiation:** The user inserts the smart card into the terminal equipment; enters the user name $Uid_{i}$, key $Upw_{i}$, and other information; and sends it to the medical service center. The medical service center carries out operations such as time verification, information finding, and verification, and then the user terminal carries out further operations and sends information to the medical service center according to the feedback from the medical service center. The medical service center carries out verification again and then interacts with the medical sensor to finally complete the verification of the user and key negotiation. The information interaction and validation steps between the entities in this process ensure that the user’s identity can be accurately authenticated and the key negotiation can be completed securely.

**User dynamic trust evaluation:** Data generated by patients using medical sensors is uploaded to the medical cloud database through the medical service center and encrypted using a specific key $K_{MSC-MCS}$. The user requests access from the medical service center and the key negotiation is securely completed. Then, the medical service center verifies the user’s key and requests access to the medical cloud database. The medical cloud database records user access behavior and dynamically adjusts the user authentication factor policy, according to which the medical service center re-authenticates medium-risk users and blocks high-risk users. This process ensures that user threats can be detected in a timely manner and appropriate response measures can be taken to ensure security.

**User authentication factor update phase:** User $U_{i}$ inputs the authentication factor update request to the user terminal while the terminal carries out relevant operations and sends information to the medical service center. The medical service center carries out time verification, information verification, and other operations and sends feedback to the user terminal. After the user terminal carries out the verification and update operations, it provides the new information to the medical service center. The medical service center performs verification and storage operations again and finally sends the updated information to the user. This ensures that the user authentication factor can be updated timely and accurately, guaranteeing the validity and security of user authentication.

**User re-authentication phase:** After receiving the updated A from the medical blockchain, the medical service center re-authenticates the online users with increased risk and locks the relevant data for uploading. After receiving the re-authentication message, the user terminal requests the user to carry out strong identity authentication, and the user carries out relevant operations and sends information to the medical service center. The medical service center carries out time verification and information verification to complete the re-authentication of the user. If the user passes the re-authentication, the medical service center releases the data access lock and sensor data upload lock and readjusts the user’s reputation. This phase ensures timely re-authentication in the event of increased risk to the user, guaranteeing the security and stability of the system.

### 4.2. Proof of Security

In this part, we will prove the proposed scheme can resist impersonation attacks, man-in-the-middle attacks, static password modification attacks, etc., by proving Theorems 1 and 2.

**Theorem 1.**  *Under particular security assumptions, the scheme proposed in this paper can withstand impersonation attacks, man-in-the-middle attacks, and static password modification attacks.*

**Theorem 2.**  *Under the stochastic prediction model, the scheme proposed in this paper can protect the anonymity of users and prevent the leakage of their private information.*

#### 4.2.1. Proof of Theorem 1

Assume that there exists a PPT attacker α who tries to perform an impersonation attack, man-in-the-middle attack, or password modification attack in the scheme of this paper. Choose the appropriate security model and assumptions, e.g., assume that attacker α cannot obtain the critical information of the user’s iris, smart card, and static passphrase.

Initialization: attacker α announces the goal of his attack, e.g., to impersonate a legitimate user for authentication or to tamper with messages during the authentication process.

Setup: The system is initialized according to the security parameters, and relevant keys and parameters are generated and distributed to legitimate users and entities. In this process, it is ensured that the generation and distribution of keys are secure and an adversary cannot obtain or tamper with these keys. The keys are generated using a secure key generation algorithm and distributed to the relevant entities over a secure channel. The system also stores and manages the keys properly to ensure their security.

Challenge: Attacker α launches an attack to try to impersonate a user, tamper with messages, or perform other malicious behavior. For example, attacker α attempts to intercept a communication message between a user and a healthcare service center and modify the authentication information in it to impersonate a legitimate user for authentication. Alternatively, an attempt is made to insert false messages or tamper with data in the smart card during the authentication process to interfere with the normal conduct of authentication. Suppose that in the authentication and key negotiation phase, the user inserts the smart card into the terminal device, obtains $\left\{Cid,MA_{i},MB_{i},UR_{UR1}\right\}$, enters the username $Uid_{i}$ and key $Upw_{i}$, generates the current timestamp $T_{6}$, and sends $\left\{T_{6},UR_{UR1}\right\}$ to the healthcare service center. The attacker tries to intercept and modify this message to send it to the medical service center by tampering with it to become $\left\{T_{6}^{\prime },UR_{UR1}^{\prime }\right\}$. The medical service center generates the current timestamp $T_{6}^{\prime \prime }$, verifies whether $T_{6}^{\prime \prime }-T_{6}^{\prime }\leq ▵T$ is valid, calculates $I_{U}=h\left(UR_{UR1}^{\prime }MSCid\right)$ if the judgment is successful, searches for table entries in the database to obtain the corresponding $\left\{T_{3},L_{i}\right\}$ with $I_{U}$ as the index, checks whether $T_{6}-T_{3}$ is expired or not, verifies whether $T_{6}-T_{3}$ is expired or not, and verifies if the value of $L_{i}$ is “3” after passing. If it is “3”, it rejects the user request, and if the verification passes, then it temporarily stores $\left\{UR_{UL},UA_{i},UB_{i},HR_{UR2}\right\}$ and generates the current timestamp $T_{7}$ and sends $\left\{T_{7},L_{i}\right\}$ to the user. At this point, due to tampering by the attacker, the information obtained by the healthcare service center is incorrect, resulting in authentication failure or the incorrect authentication of the attacker.

Response: The system responds to and authenticates the behavior of attacker α according to the designed security protocol. During the authentication process, the system verifies the authentication factors using pre-stored user information and keys to ensure that the identification factors match the user’s identity. If an anomaly is detected, the system will reject the authentication request and take appropriate security measures, such as recording the attack behavior and issuing an alert.

If attacker α can successfully break through the security defense of the system, i.e., win the security challenge of the attack with a non-negligible advantage, then it means that there is a security vulnerability in the system. However, according to the scheme design, by combining the three-factor authentication with iris recognition, smart cards, and static passwords, as well as the re-authentication mechanism, it can effectively prevent impersonation attacks, man-in-the-middle attacks, password modification attacks, etc.

The uniqueness and difficulty in counterfeiting iris factors make it difficult for attackers to impersonate the iris characteristics of legitimate users. Iris recognition technology usually has high accuracy and security and can effectively distinguish the iris features of different users. The secure storage and encryption features of smart cards ensure that the data in the smart card cannot be easily stolen or tampered with. Smart cards can store important data such as users’ authentication information and keys and protect these data through encryption algorithms, which can only be decrypted and accessed by having the correct key. The encrypted transmission and verification of the static passphrase increases the difficulty for the adversary to obtain the static passphrase. The static passphrase is encrypted during transmission and is verified by comparing it with the hash value stored in the system to ensure the accuracy of the passphrase. In addition, the re-authentication mechanism further enhances the security of the system, guaranteeing that only legitimate users can pass the authentication. The re-authentication mechanism further ensures the legitimacy of the user’s identity by enforcing the re-authentication of untrustworthy users in the case of abnormal user dynamic trust evaluation.

Therefore, if attacker α can win the security challenge of the attack with a non-negligible advantage, then this contradicts the scheme design and security mechanism, thus proving that the scheme put forward in this paper can resist impersonation attacks, man-in-the-middle attacks, and password modification attacks.

#### 4.2.2. Proof of Theorem 2

Assume that there exists an attacker β who tries to break the anonymity of the user. We will play a security game with attacker β. The prerequisite for winning the security game is that attacker β can successfully identify the real identity of the user.

Initialization: attacker β selects a target user and tries to obtain information related to that user.

Query: attacker β submits a query request to the system and tries to obtain the user’s identification information or other relevant data.

Challenge: The system responds to attacker β’s query according to the security policy, providing only the necessary anonymized information to ensure that the user’s anonymity is protected. Assume that the system encrypts the user’s sensitive information using a cryptographic function $E\left(\cdot \right)$, e.g., the user’s identifier ID is encrypted as $E\left(ID\right)$. At the same time, the system uses the anonymized identifier AID instead of the user’s real identity information.

Guessing: Assume that the information obtained by attacker β includes the encrypted user’s sensitive information $E\left(ID\right)$ and the anonymous identifier AID. Attacker β tries to guess the user’s real identity $ID^{\prime }$ based on this information.

If attacker β wins the game in the guessing phase with a non-negligible advantage and succeeds in identifying the user’s real identity, i.e., $ID^{\prime }=ID$, it implies that there is a vulnerability in the anonymity protection mechanism of the system. However, according to the design of our scheme, by using encryption and anonymization, the user’s identity information is effectively protected during the authentication process, and attacker β cannot accurately identify the user’s true identity even with the information they’ve obtained. Therefore, under the random prediction model, no attacker β can break the user anonymity of this paper’s scheme with a non-negligible advantage.

### 4.3. Security and Privacy Protection

In this section, we analyze the advantages of the proposed scheme from four different aspects (i.e., privacy protection, data availability and trustworthiness, confidentiality, and resilience).

**Data availability and trustworthiness:** In our proposed protocol, user authentication factors have dynamic attributes. First-time logged-in users need only a smart card and static password for convenience. For security, we use iris biometric authentication for secondary confirmation for high-risk users. Users with elevated risk may be accessing infrequently accessed resources, logging in and out too many times, have unstable data interactions with medical sensors, exhibit abnormal behavior or dangerous access, etc., resulting in a rapid decline in the user’s trust value, which can result in the downgrading of access privileges and the requirement of the re-authentication of the user’s identity. To link the dynamic trust evaluation to real-world applications and validate it, we integrate real-world data. Analyzing historical data from similar medical systems helps set trust thresholds. For example, we define “infrequent access” based on real-world resource access frequencies. All the stored data in this scheme are ciphertext data encrypted by the SM3 hash function, and according to the unidirectional character of the SM3 hash function, all the information can not be obtained by backpropagation. The final tripartite key $K=h\left(T_{11}R_{UL1}\cdot R_{UL2}\cdot R_{UL3}\cdot P\right)$ can only be calculated by the user, medical service center, and medical sensor. The blinded user and sensor IDs at the medical service center prevent attackers from correlating obtained information, balancing authentication usability and security. At the same time, when the user is identified as a high-risk user, the medical service center cancels the account and locks it. The user can re-register but not access previous info. Lost or stolen smart cards can be invalidated by re-registration. To address the single smart card security risk for authentication and data storage, we introduce a backup mechanism. In the case of a lost or compromised smart card, users can use a temporary code sent to their trusted mobile devices to access essential medical data. This scheme offers good usability and ensures secure access to information resources in emergencies.

**Confidentiality:** In the registration and authentication phase, the communication between the medical service center and the medical blockchain and between the user and the medical service center is securely encrypted. During the key generation process, the relevant information is subjected to hashing using the SM3 algorithm to generate a high-strength encryption key. This ensures that the two communicating parties can share the key securely. When encrypting messages, by combining the hashing operation of SM3 with other encryption algorithms, the encrypted messages become extremely difficult to crack during transmission, thus guaranteeing the confidentiality of the communication. In addition, user data and medical sensor data in the medical cloud database are encrypted and stored so that only users with the appropriate key can access and read the data, ensuring the confidentiality of data storage.

**Resilience:** Blockchain network resilience is vital for continuous telemedicine services. Any disruption to the blockchain network could potentially lead to serious consequences for patient care. The Fabric consortium blockchain employed in our system has several key features to safeguard against attacks and failures. First, our Fabric consortium blockchain offers key protections. Distributed ledger tech, with each block’s hash linked to the previous one, thwarts data manipulation. In telemedicine, any attempt to alter patient records will be revealed by hash changes, maintaining data integrity. Second, smart contracts enforce access control, detecting abnormal access patterns (e.g., signs of a DDoS attack) and blocking attackers while allowing legitimate use. Moreover, the network has multiple nodes. If a node fails (due to an attack or other reasons), others keep running as data are replicated, preventing a single point of failure. Requests are rerouted to healthy nodes for continuous service. For disasters, the medical service center regularly backs up blockchain data off-site. In the case of major security issues, the network can be restored from the backup. A hot standby mechanism for nodes reduces downtime when a node fails. These measures ensure network resilience, safeguarding against threats and enabling stable telemedicine, protecting patient data and system longevity.

**Privacy protection:** In this scheme, during key negotiation, the user, medical service center, and medical sensor generate random numbers. The final session key *K* is derived using the SM3 hash function. A new session key is negotiated for each communication connection. Due to the unidirectional nature of SM3 and its avalanche effect (slight input changes cause large hash value changes) and its resistance to hash collision attacks, attackers cannot backtrack to obtain information even if they obtain the current session key. In terms of attack resistance, so far, no serious collision attack vulnerabilities have been found in the SM3 hash function. It can effectively resist common hash collision attacks, ensuring the security of tokens and credentials generated based on it, and further guaranteeing the reliability of user identity authentication and data privacy protection. To balance privacy and dynamic trust evaluation, we use a multi-tiered approach. We anonymize user data in trust evaluation, limit access to necessary information when a user’s risk level rises, and conduct iris-based re-authentication in a privacy-compliant manner. Second, the three-factor authentication scheme using iris recognition, smart cards, and static passwords increases the security of authentication and reduces the risk of user identity impersonation. To further protect user data during registration and authentication, we added additional security measures. Before registration, we use multi-step verification for identity authenticity. During authentication, we check user-provided info and data transmission channels in real time. Biometric info (like iris features) is encrypted during collection and transmission and used sparingly. Strict iris-based authentication for managers and untrustworthy users protects biometric privacy.

### 4.4. Functional Analysis

In this section, we will conduct performance test experiments on our proposed authentication scheme, and Table 3 shows the system environment in which we conduct the tests.

We analyze the security aspects of the scheme in this paper in comparison with those of An et al. [9], Chen et al. [14], Das et al. [15], Wu et al. [16], Challa et al. [17], Ali et al. [20], and Gupta et al. [21]. The comparison outcomes are shown in Table 4.

There is a difference between this paper’s scheme and An et al.’s scheme in terms of mutual authentication; An et al. and Challa et al.’s scheme cannot maintain mutual authentication, while this paper’s scheme has been improved and optimized in this aspect. In terms of password change, the schemes of An et al., Chen et al., and Ali et al. cannot maintain the security of password change, while the scheme in this paper solves this problem effectively. For user anonymity, the schemes of An et al., Das et al., Wu et al., and Challa et al. cannot maintain user anonymity, whereas the scheme in this paper ensures the protection of user anonymity by using encryption and anonymization. In terms of impersonation attacks, man-in-the-middle attacks, and password modification attacks, the schemes of An et al., Chen et al., Das et al., and Wu et al. have security concerns, whereas this paper’s scheme effectively defends against these attacks by combining three-factor authentication with iris recognition, smart cards, and static passphrases, as well as a re-authentication mechanism. In terms of parallel session attacks, the schemes of Chen et al., Challa et al., and Ali et al. are insecure, while this paper’s scheme enhances and prevents them. The schemes of Chen et al., Das et al., Wu et al., and Gupta et al. need to be revised in smart card/smart device theft attacks, whereas this paper’s scheme mitigates this risk through various security measures. In conclusion, compared to other schemes, this paper provides several functional features and is effective in preventing possible known attacks.

### 4.5. Performance Analysis

This part makes a comparison of the performance of the scheme put forward in this paper and that of other related schemes, such as those of An et al. [9], Chen et al. [14], Das et al. [15], Wu et al. [16], Challa et al. [17], Ali et al. [20], and Gupta et al. [21] based on the security and functional characteristics and computational and communication costs of message transmission in the login and authentication phases. This assessment enables us to have a clearer understanding of the superiority of the scheme in this paper compared to other schemes.

#### 4.5.1. Comparison of Communication Costs

Table 5 presents a comparison of the communication overhead between our scheme and several related ones. For the purpose of comparing communication costs, the following assumptions are made: the bit lengths needed for identity, random numbers, timestamps, authentication (signatures via the Elliptic Curve Digital Signature Algorithm (ECDSA)), hash results (assuming SHA-1 as $h\left(\cdot \right)$), and Message Authentication Codes (MACs) are 64, 64, 64, 320, 160, and 160 bits, respectively. Additionally, to ensure adequate security, 1024 bit modes are employed for both the modulo power-taking and inversion operations in this comparative analysis.

Under these considerations, the An et al.’s scheme has a message count of three and a communication overhead of 864 bits; the Chen et al.’s scheme has a message count of two and a communication overhead of 736 bits; the Das et al.’s scheme has a message count of three and a communication overhead of 1280 bits; the Wu et al.’s scheme has a message count of six and a communication overhead of 1152 bits; the Challa et al.’s scheme has a message count of three and a communication overhead of 1984 bits; the Ali et al.’s scheme has a message count of three and a communication overhead of 1400 bits; the Gupta et al.’s scheme has a message count of seven and a communication overhead of 2144 bits; and the scheme of this paper has a message count of three and a communication overhead of 712 bits. Comparing all these overheads, the communication overhead of this paper’s scheme turns out to be the most favorable. Our scheme is able to efficiently withstand a variety of attacks, guarantee the security of user information, and supply more comprehensive functions to satisfy the demands of telemedicine.

#### 4.5.2. Comparison of Calculation Costs

In our computational cost analysis, performance metrics are taken into account, as presented in Table 6.

Table 7 further conducts a comparative analysis of the computational cost during the login and authentication phases, verifying the superiority of our scheme over others. Based on the experimental findings in Table 7, the computational time demanded by our scheme, along with those of An et al., Chen et al., Das et al., Wu et al., Challa et al., Ali et al., and Gupta et al. are 0.0048 s, 0.00584 s, 0.09542 s, 0.01024 s, 0.07448 s, 0.01902 s, and 0.01870 s, respectively. It is evident from Table 7 that while our scheme incurs slightly more computational cost compared to An et al.’s, the latter offers relatively rudimentary security functions. For example, it is weak in resisting certain specific attacks (such as impersonation attacks, man-in-the-middle attacks, etc.) or ensuring user anonymity and password change security. In contrast, our proposed scheme is reasonable since it provides additional functional features and better security than the other schemes (see Table 4).

## 5. System Evaluation

### 5.1. Test Environment

In this section, based on authoritative standards including international and national standards, this paper conducts experimental tests on the proposed solution. It performs a performance test on the blockchain system employed in the solution to determine whether the system performance meets the requirements. The testing software used is Hyperledger Caliper 0.5.0, and the test environment is presented in Table 8.

### 5.2. Test Basis

In this section, referring to relevant blockchain benchmark documents, the performance test indicators of the blockchain are sorted out, as shown in Table 9. The benchmark documents referred to are as follows:

- Hyperledger Blockchain Performance Metrics [27].
- Information security technology–security framework for blockchain technology [28].

### 5.3. Results and Analysis

Hyperledger Caliper serves as a benchmark framework for blockchain performance. It produces test reports in the format of HTML documents and currently has the capacity to evaluate performance metrics like the success rate and resource consumption. In a high-concurrency and high-load environment, we utilize Caliper to assess the performance of our blockchain system. The specific functions under test are the chaincode function “invoke” for uploading data to the ledger and the chaincode function “query” for querying the ledger. During the testing process, we monitor in real time the number of transactions received per second and the number of transactions processed per second by the blockchain system. We then regulate the sending pressure to ensure that these two values are approximately the same, thereby pushing the pressure to reach the system’s bottleneck. Subsequently, by increasing the frequency of transaction requests, the number of transactions received per second by the system becomes significantly higher than the system’s transaction throughput. This causes the system to operate under extreme conditions, which is precisely the objective of the spike test.

The test statistics are shown in Table 10.

For the invoke smart contract, three tests were conducted in this section, with 1000 transactions sent in each test. Regarding the query smart contract, two tests were carried out, and 5000 transactions were sent in each test. As a result, the total number of transactions sent was 13,000, with 3000 for function calls (from the invoke contract tests) and 10,000 for queries (from the query contract tests). As can be seen from Table 10, the number of successful transactions for queries is equal to the total number of transactions they sent. Therefore, the count of failed transactions is 0. The test results show that all performance indicators meet the requirements and the baseline.

## 6. Conclusions

In the field of telemedicine, with the continuous evolution of the service model, the convenience of obtaining medical services remotely also causes problems such as complex user access authentication, easy leakage of user privacy, and a lack of comprehensive consideration of access control. In this regard, this paper proposes an identity management scheme based on multi-factor authentication and dynamic trust evaluation for telemedicine. The scheme adopts a three-factor authentication method combining iris recognition, smart cards, and static passwords, and incorporates a dynamic trust evaluation mechanism based on behavioral analysis to achieve accurate authentication and dynamic trust evaluation of user identity. The security analysis ensures the solution can effectively defend against some common security threats. In addition, it protects users’ anonymity and private information from leakage through encryption technology and anonymization. The experimental findings indicate that our scheme holds performance superiority compared to the existing authentication protocols. System evaluation based on authoritative standards was conducted, and the results demonstrated that all performance indicators of the proposed scheme met the requirements and baseline, further validating the feasibility and reliability of this scheme in practical applications. The dynamic trust evaluation mechanism employs decision trees or neural network algorithms to accurately classify and dynamically adjust user trust levels based on key indicators such as the malicious attack rate, attempted privilege escalation rate, abnormal login system rate, and duration of service access. In the identity authentication process, iris recognition devices, smart cards, and static passwords work in close coordination under the efficient management of the software system, ensuring the accuracy and efficiency of the authentication process. At present, due to the lack of sufficient actual data to test, this paper lacks sufficiently fine-grained access control for the actual accesses carried out by users, and further research is still needed to optimize and improve this scheme in the future. The next steps are as follows:

- Upgrade with advanced biometric algorithms or smart card encryption technologies to optimize the authentication process, shorten the authentication time, reduce user operation steps, enhance the efficiency of telemedicine identity authentication, and improve the user experience;
- Expand dynamic trust evaluation indicators, analyze user behavior in depth, and enhance the adaptability of the dynamic trust evaluation model to achieve a more accurate and comprehensive risk assessment.

## Figures and Tables

**Table 1:** Notations and cryptographic functions.

Symbols	Description
$U_{i}$	User *i*
$Uid_{i}$	$U_{i}$’s account
$Upw_{i}$	Login key for *i*
$Upw_{i}$	Iris characteristics of *i*
$h\left(\cdot \right)$	Hash function
⊕, ∥	Bitwise XOR and concatenation operations, respectively
Gen(·)	Probabilistic generation in fuzzy extractor
Rep(·)	Deterministic reproduction procedures in fuzzy extractor
$\sigma_{i}$ $\tau_{i}$	Biometric secret key and public reproduction parameter, respectively
▵T	Maximum transmission delay
$L_{i}$	User authentication factor policies regularly updated from healthcare cloud servers
SK	Common session key

**Table 2:** Classification of trust levels.

Trust Level	Trust Interval	Character Set	Authentication Methods
Untrustworthy	[0,30]	High-risk user	Denial of login
Basic trustworthy	[30,60]	Medium-risk users	Re-authenticate
General trustworthy	[60,80]	Regular user	Smart card authentication method
High trustworthy	[80,100]	Low-risk user	Any authentication method

**Table 3:** System environment.

Item	Parameter
Operating system	Windows 10 64 bit
Processing unit	15 vCPU Intel(R) Xeon(R) Platinum
	8338C CPU @ 2.60 GHz
Random access memory (RAM)	80 GB
GPU	RTX 3090(24 GB)
Hard disk capacity	75 GB
Experimental platforms	Pycharm 2024.1.1

**Table 4:** Security function analysis.

Security Attribute	An et al. [9]	Chen et al. [14]	Das et al. [15]	Wu et al. [16]	Challa et al. [17]	Ali et al. [20]	Gupta et al. [21]	Ours
Mutual authentication	×	*√*	*√*	*√*	×	*√*	*√*	*√*
Key agreement	*√*	×	*√*	*√*	*√*	×	*√*	*√*
Password change	×	×	*√*	*√*	*√*	×	*√*	*√*
User anonymity	×	*√*	×	×	×	*√*	*√*	*√*
Attack by impersonation	×	×	×	*√*	*√*	*√*	*√*	*√*
Man-in-the-middle attack	×	×	*√*	*√*	*√*	*√*	*√*	*√*
Password change attack	×	×	×	×	*√*	*√*	*√*	*√*
Parallel session attack	*√*	×	×	*√*	×	×	*√*	*√*
Smart card/smart device theft attacks	*√*	×	×	×	*√*	*√*	×	*√*

**Table 5:** Communication overhead during the login and authentication phase.

Scheme	Number of Messages	Communication Overhead (bit)
An	3	864
Chen	2	736
Das	3	1280
Wu	6	1152
Challa	3	1984
Ali	3	1400
Gupta	7	2144
ours	3	712

**Table 6:** Approximate time required for various operations.

Operational Time Costs	
$T_{mul}$: time for modulo multiplication	0.00088 s
$T_{e}$: execution time of Elliptic Curve dot product operations	0.0171 s
$T_{h}$: execution time of the hash function	0.00032 s
$T_{fe}$: function execution time of the fuzzy extractor	0.0171 s

**Table 7:** Comparison of calculated costs.

Scheme	Cost	Time (s)
An	15$T_{h}$	0.00480
Chen	10$T_{h}$ + 3$T_{mul}$	0.00584
Das	31$T_{h}$ + 4$T_{e}$ + $T_{fe}$	0.09542
Wu	32$T_{h}$	0.01024
Challa	19$T_{h}$ + 3$T_{e}$ + $T_{fe}$	0.07448
Ali	6$T_{h}$ + $T_{e}$ + $T_{fe}$	0.01902
Gupta	7$T_{h}$ + $T_{e}$	0.01870
Ours	9$T_{h}$ + 3$T_{mul}$	0.00552

**Table 8:** Test environment.

Item	Server-Side Environment	Client-Side Environment
Hard disk	1TB SSD drive	512G SSD hard drive
Bandwidth	200 Mbps	200 Mbps
Operation system	Ubuntu 20.04 LTS	Windows 10 64 bit
Blockchain system	Hyperledger Fabric 2.4.9	N/A

**Table 9:** Blockchain performance metrics.

Rule Item	Requirement
Transaction latency	TPS ^a^ and TRS ^b^ should be approximately equal.
Transaction throughput	The system should operate under high load without crashing.
Performance indicator	The average response time should be less than 0.5 s, with a transaction success rate higher than 95% and TPS higher than 200.
Spike testing	TRS should be significantly higher than TPS, with a transaction success rate higher than 80%.

^a^ “Transactions processed per second” is the abbreviation for TPS. ^b^ “Transactions received per second” is the abbreviation for TRS.

**Table 10:** Test statistics.

Performance Indicator	Invoke Function	Query Function
Transaction success number (pens)	3000	10,000
Transaction sending rate	341	359
Transaction throughput	203	347
Maximum transaction latency (s)	5.03	4.21
Minimum transaction Latency (s)	1.26	0.01
Average transaction Latency (s)	2.98	1.03

## Data Availability

Data related to this study are available and can be obtained by contact ing the corresponding author.
